# Highly magnetic hybrid foams based on aligned tannic acid-coated iron oxide nanoparticles and TEMPO-oxidized cellulose nanofibers†

**DOI:** 10.1039/d3ra01896b

**Published:** 2023-05-09

**Authors:** Seyed Ehsan Hadi, H. Aygül Yeprem, Agnes Åhl, Mohammad Morsali, Martin Kapuscinski, Konstantin Kriechbaum, Mika H. Sipponen, Lennart Bergström

**Affiliations:** a Department of Materials and Environmental Chemistry, Stockholm University Stockholm 10691 Sweden lennart.bergstrom@mmk.su.se; b Wallenberg Wood Science Center, Department of Materials and Environmental Chemistry, Stockholm University Stockholm 10691 Sweden; c Department of Metallurgical and Materials Engineering, Yıldız Technical University Istanbul 34220 Turkey

## Abstract

Lightweight iron oxide nanoparticle (IONP)/TEMPO-oxidized cellulose nanofibril (TOCNF) hybrid foams with an anisotropic structure and a high IONP content were produced using magnetic field-enhanced unidirectional ice-templating. Coating the IONP with tannic acid (TA) improved the processability, the mechanical performance, and the thermal stability of the hybrid foams. Increasing the IONP content (and density) increased the Young's modulus and toughness probed in compression, and hybrid foams with the highest IONP content were relatively flexible and could recover 14% axial compression. Application of a magnetic field in the freezing direction resulted in the formation of IONP chains that decorated the foam walls and the foams displayed a higher magnetization saturation, remanence, and coercivity compared to the ice-templated hybrid foams. The hybrid foam with an IONP content of 87% displayed a saturation magnetization of 83.2 emu g^−1^, which is 95% of the value for bulk magnetite. Highly magnetic hybrid foams are of potential interest for environmental remediation, energy storage, and electromagnetic interference shielding.

## Abstract 2

The use of both magnetic field-enhanced unidirectional ice-templating (MFUIT) and unidirectional ice-templating (UIT) techniques of dispersions can lead to the production of highly magnetic hybrid anisotropic foams with a high amount of IONP.

## Graphical abstract

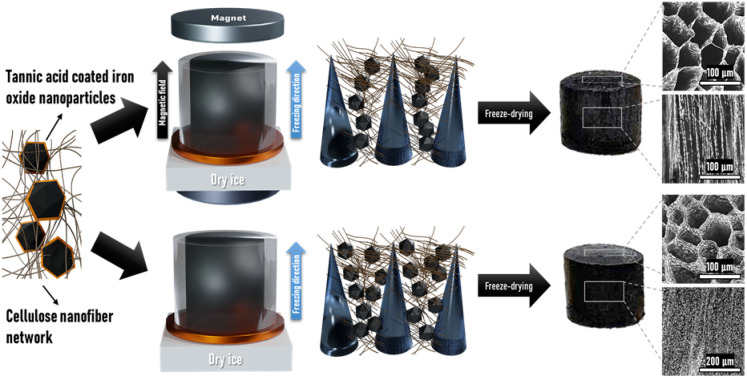

## 1. Introduction

Lightweight and highly porous hybrid foams based on iron oxide nanoparticles (IONP) together with polymeric or fibrillar components that are both magnetic and mechanically robust are of interest for, *e.g.*, cleaning of oil spills, thermal insulation, and high-performance supercapacitors.^1–4^ Synthetic polymers such as polyurethane and poly(vinyl alcohol), as well as chitosan and cellulose, have been used to fabricate lightweight IONP/polymer foams or aerogels.^1,3,5^ The polymers promote the formation of a mechanically stable three-dimensional foam or aerogel structure with the incorporated magnetic nanoparticles. The mechanical stiffness and flexibility, high surface area, and versatile surface chemistry of nanocellulose have motivated a large amount of research activity to utilize this nanofibrillar and hydrophilic material as a scaffold or matrix to generate nanocomposite magnetic foams.^6–11^ One of the challenges in the fabrication of cellulose nanofiber (CNF)-based magnetic foams is preventing uncoated IONP aggregation, particularly in high amounts of IONP, which can compromise the mechanical and magnetic properties of the foams.^2,12^ To build a more stable network, an intermediate compound that can bind to both CNF and IONP could be used. Tannic acid (TA) is a naturally occurring polyphenol with a high affinity for both CNF and IONP. TA is expected to form coordination bonds with metal ions in metal oxide nanoparticle defects and dangling bonds on its surface, as well as hydrogen bonds with CNF, allowing it to efficiently bind to CNF and coat IONP.^13–17^ The magnetic hybrid foams are of potential interest for a range of applications, including microwave absorption, environmental remediation, and energy storage.^5^ Recent reports include isotropic CNF-based foams containing up to 15 wt% IONP for thermal insulation^2^ and isotropic nanocellulose-IONP foams with a density of 9.2 mg cm^−3^ and maximum IONP content of 0.8 wt% for oil adsorption applications.^18^ He *et al.* demonstrated a hybrid isotropic foam with a density of 29 mg cm^−3^ composed of CNF, polylactic acid, carbon nanotubes and a maximum IONP content of 2 wt% that displayed a saturation magnetization value of 10.1 emu g^−1^.^19^ Srasri *et al.* reported cellulose-based aerogels with an IONP content up to 30% for dye adsorption^20^ and Wei *et al.* demonstrated a cellulose-based/IONP aerogel with saturation magnetization of 53.7 emu g^−1^ for adsorption of Cr(vi) ions from aqueous solutions.^21^ The saturation magnetization of the hybrid foams is mainly determined by the amount of IONP present in the hybrid foams^22^ but increasing the amount of IONP beyond 30–40 wt% is rarely reported as high IONP content can compromise the mechanical and structural integrity of the hybrid foams.

In this work, anisotropic IONP/TA/TEMPO-oxidized CNF (TOCNF) magnetic foams with an IONP content of up to 87 wt% were fabricated by combining unidirectional ice-templating with magnetic alignment. Tannic acid (TA) was used to improve the colloidal stability and processability of IONP and TOCNF, and to improve the mechanical properties of the resulting hybrid foams. The synergistic action of directional growth of ice crystals and magnetic alignment, and chain-formation of IONP resulted in flexible foams with a well-defined cellular structure and a maximum magnetic saturation of 83.2 emu g^−1^, which corresponds to 95% of the value for bulk magnetite.

## 2. Experimental

### 2.1. Materials

Never-dried sulfite softwood cellulose pulp, also known as Domsjö dissolving Plus, was procured from Domsjö Fabriker AB (Aditya Birla Domsjö, Sweden) and carefully treated with HCl_(aq)_ with the pH of 2. TEMPO (2,2,6,6-tetramethyl-1-piperidinyloxy) (Sigma Aldrich), iron oxide nanoparticles (iron(ii,iii) oxide, 50–150 nm, Sigma Aldrich), sodium bromide (NaBr, Sigma Aldrich), tannic acid (TA, Alfa Aesar), sodium hypochlorite (NaClO, Merck), hydrochloric acid (HCl ∼35%, VWR international), and sodium hydroxide (NaOH ≥99.2%, VMR international) used as received. Unless otherwise noted, all studies were conducted using deionized water.

### 2.2. Preparation of TOCNF dispersion

TOCNF was prepared using the procedure reported by Isogai *et al.*^23^ Briefly, to produce TOCNF, 40 g of never-dried softwood sulfite cellulose pulp was treated with 10 mmol of NaClO per gram of cellulose at a pH of 10 for 200 minutes. To remove any remaining chemicals, the oxidized pulp was thoroughly rinsed with deionized water. The Masuko Sangyo Co., Ltd Model MKZA10-15 J supermasscolloider grinder, equipped with non-porous grinding stones, was used to carry out the grinding process for the production of TOCNF. Furthermore, the oxidized pulp was diluted to 1 wt% before being passed through the supermasscolloider two to three times at a rotating speed of 25 Hz and a gap distance of −100 μm.

### 2.3. Preparation of IONP/TA dispersion

82.5 mg of TA with specified amount of IONP and 1 ml of 0.1 M NaOH were added to 6 ml of water. The resulting dispersion was then sonicated using a sonicator (Sonics VCX-130, USA) with a 6 mm probe at an intensity of 75% for 30 minutes to produce a homogeneous IONP/TA dispersion.

### 2.4. Preparation of mixed TOCNF and IONP or IONP/TA dispersions

IONP/TOCNF and IONP/TA/TOCNF dispersions were prepared by mixing specified amounts of the IONP or TA/IONP and 16.5 g of TOCNF stock dispersions (1 wt%) at a pH of 7 under continuous sonication, respectively. Water was added to the resulting dispersions to dilute them to a TOCNF concentration of 0.5 wt% and then they were vigorously vortexed for 30 minutes.

### 2.5. Preparation of foams

**Fig. 1:**
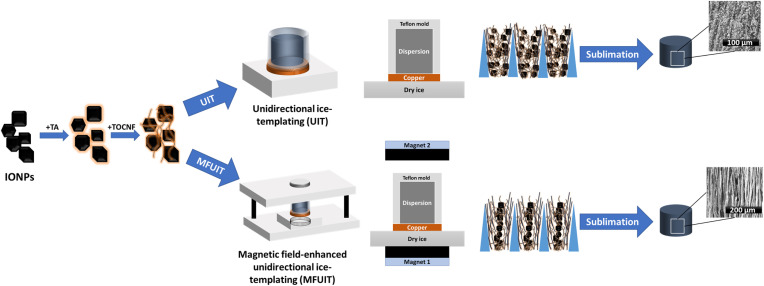
Schematic illustration of the processing of anisotropic magnetic hybrid foams based on iron oxide nanoparticles (IONP), tannic acid (TA) and TEMPO-oxidized cellulose nanofibrils (TOCNF) by UIT and MFUIT.

TOCNF and TOCNF/TA foams were prepared by unidirectional ice-templating (UIT) of aqueous dispersions (Fig. 1). Briefly, 5 g of recently vortexed dispersions were poured into a cylindrical Teflon mold with a diameter of 2 cm and a height of 2 cm closed by a removable copper bottom plate. The sample-holding mold was then placed on dry ice (−78.5 °C) until the directional freezing of the dispersions was completed. The frozen dispersions were carefully removed from the mold and freeze-dried for a minimum of 48 hours (Alpha 1-2 LDplus, CHRIST, Germany). Anisotropic IONP/TA/TOCNF and IONP/TOCNF foams were prepared by both magnetic field-enhanced unidirectional and unidirectional ice-templating of dispersions. Briefly, 5 g of recently vortexed dispersions were poured into a cylindrical Teflon mold with a diameter of 2 cm and a height of 2 cm closed with a removable bottom copper plate. For magnetic field-enhanced unidirectional ice-templating, the sample-holding mold was then subjected to a directional and homogeneous magnetic field (with the strength of 35 mT at the center of the mold) generated by two identical magnets with known properties. While the dispersion was still exposed to the magnetic field, a directional thermal gradient is carefully applied to the mold by placing the sample-holding mold on dry ice (−78.5 °C) until the directional freezing of the dispersion was finished. The same process was followed for unidirectional ice-templating without any magnetic field being applied. Structured frozen dispersions were then carefully removed, and the final dry foam was obtained by placing the frozen dispersions for a minimum of 48 hours in a freeze-dryer (Alpha 1-2 LDplus, CHRIST, Germany).

### 2.6. Characterization

The surface charge of the TOCNF was determined to be 1.1 mmol COO^−^ per g utilizing conductometric titration with sodium hydroxide as a titrant. The tabletop TM 3000 (Hitachi High Tech, Japan) with an accelerating voltage of 15 kV equipped with a backscattered electron detector was used to investigate the structure of the foams. The distribution of IONP on the walls of foams was investigated using a JSM-7400F (JOEL Ltd, Japan) scanning electron microscope (SEM) with a secondary electron detector and a 15 kV accelerating voltage. The foams were sliced both horizontally and vertically while they were frozen. Transmission electron microscopy (TEM) images were captured with a JEM-2100F (JOEL Ltd, Japan) at a 200 kV acceleration voltage. A drop of dispersion with 0.005% concentration was put over carbon-coated copper and air dried. Atomic force microscopy (AFM) images were taken using a Multimode-8 AFM (Bruker, USA) in peak-force tapping mode with the ScanAsyst™ automatic optimization algorithm. A drop of dispersion with 0.005% concentration was placed on freshly cleaved mica and air dried. The zeta potential was measured using a Zetasizer Nano ZS90 device (Malvern Instruments Ltd, UK) with a dip cell probe. The pH of the dispersions was adjusted using aqueous hydrochloric acid and sodium hydroxide solutions. Fourier-transform infrared spectroscopy (FT-IR) measurements were carried out on a Varian 610-IR FTIR spectrometer with a resolution of 2 cm^−1^ ranging from 450 cm^−1^ to 4000 cm^−1^. All the samples were measured in their dry powder state. After sonicating TA/IONP for 30 minutes, the coated IONP were collected with a magnet and thoroughly cleaned with water. After air drying the wet coated IONP for 30 minutes at 60 °C, they were FTIR characterized.

The rheological characteristics of dispersions were examined using a Physica MCR 301 rheometer with a concentric cylinder (CC27, 27 mm diameter cylinder) (Anton Paar GmbH, Austria). To replicate the condition of dispersions at the time of the process, all samples were vortexed vigorously for 5 minutes prior to measurements and then subjected to a pre-shear at a shear rate of 1000 s^−1^ for 2 minutes right before each measurement. The amplitude and frequency sweep measurements were performed at 10 rad s^−1^ angular frequency and 0.1% strain, respectively. For the three interval thixotropy test (3ITT), samples were sheared at a constant rate of 10 s^−1^ for 120 seconds, then at a constant rate of 1000 s^−1^ for 30 seconds, and finally at a constant rate of 10 s^−1^ for 600 seconds. The recovered viscosity values for each dispersion were reported 30 seconds after finishing shear at a constant rate of 1000 s^−1^. All measurements were carried out at a temperature of 25 °C. By using the vibrating sample magnetometer (VSM) option of PPMS (Quantum Design, USA), the magnetic moments of the magnetic foams were calculated as a function of the applied DC magnetic field perpendicular to the long axis (alignment direction) of the foams. The samples were subjected to a magnetic field in the range of −2 T to 2 T at a temperature of 300 K. The mechanical performance of foams was investigated through the execution of a compression test. This test was conducted on an Instron 5966 universal testing machine (Instron, USA) equipped with a 100 N load cell. All the foams were conditioned for at least 48 hours prior to testing at 23 °C and 50% RH. The dimensions and weight of the foams were measured with a digital caliper and an analytical balance, respectively, and their apparent density was calculated prior to measurement. The strain rate for all measurements was set to 10% per minute. The slope of the initial linear segment of the stress–strain curve was utilized in the computation of Young's modulus. Furthermore, the calculation of toughness was conducted by determining the area beneath the stress–strain curve up to the final measurement point. The average of 4 measurements with one standard deviation per sample was reported. Thermogravimetric analysis of foams was performed using a Discovery TG (TA Instruments, USA). All experiments were carried out in a nitrogen flow at a rate of 25 ml min^−1^. Temperatures ranged from 30–800 °C, with heating rates of 10 °C min^−1^. The surface area of the bare IONP was analyzed using the Brunauer–Emmett–Teller (BET) model based on the N_2_ adsorption/desorption isotherm. The measurement was performed at −196 °C using a Micrometrics ASAP 2020 instrument. Prior to analysis, the sample had been degassed at 105 °C for 12 hours.

## 3. Results and discussion

Hybrid IONP/TOCNF foams were prepared using both conventional unidirectional ice-templating (UIT) and magnetic field-enhanced unidirectional ice-templating (MFUIT) (Fig. 1). The anisotropic hybrid foams were prepared from aqueous dispersions of TOCNF and commercially available iron oxide nanoparticles that were stabilized with TA. The optimization of the colloidal properties of TA-coated iron oxide nanoparticles and TOCNF enabled the preparation of hybrid foams with very high IONP content and structural integrity (Fig. 1). The proportion of TA and IONP in the dispersions varied while the TOCNF content of all dispersions remained constant at 0.5 wt%. The composition of the dispersions (*D*) and foams (*F*_UIT_ or *F*_MFUIT_) is identified by three numbers that correspond to the relative amount of IONP, TA and TOCNF, respectively. The “*D*(2.5 : 0.5 : 1)” dispersion, for example, contains 62.5 mg of IONP, 12.5 mg of TA, and 25 mg of TOCNF, with a total solid mass of 100 mg. The foam that was produced from this dispersion with the UIT technique is thus “*F*_UIT_(2.5 : 0.5 : 1)” and the corresponding foam fabricated with the MFUIT technique is abbreviated “*F*_MFUIT_(2.5 : 0.5 : 1)”.

**Fig. 2:**
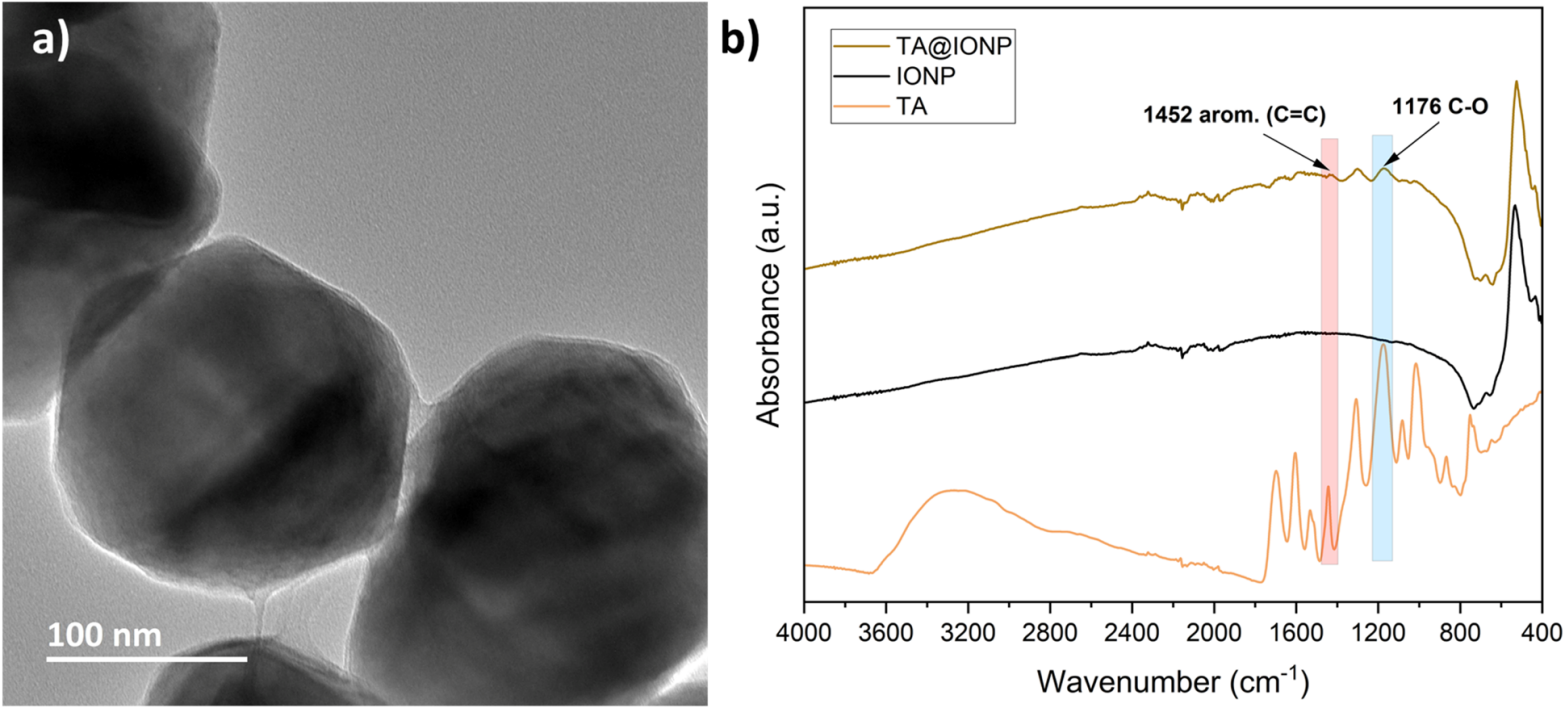
Characterization of TA-coated IONP (TA@IONP). (a) TEM image of TA@IONP. (b) FTIR spectrum of TA@IONP, IONP, and TA. Red and blue marked region belongs to aromatic in-ring (C–C) stretch and (C–O) stretch, respectively.

The TOCNF used in this study had an average length of 460 nm ± 208 nm, as determined by peak-force tapping mode AFM micrographs of 50 particles (Fig. S1a†). The surface charge of TOCNF was estimated to be 1.1 mmol g^−1^ and the zeta potential is −50 mV ± 5 mV at a pH of 7 and 25 °C. The IONP used in this study have an average size of 195 nm ± 11 nm as determined from TEM images of 20 particles (Fig. 2a). The IONP displayed a zeta potential of −20 mV ± 2 mV at a pH of 7 and 25 °C, and were colloidally unstable. The addition of TA to the aqueous IONP dispersion reduced the zeta potential at a pH of 7 to −38 mV (Fig. S2†), which indicates that TA adsorbed onto the IONP surface. The TEM image (Fig. 2a) also indicates that the IONP is covered with a thin layer that forms a meniscus at the contact point of two particles. Previous works have demonstrated that TA can effectively bond to CNF and coat IONP.^13–15,24^ The presence of peaks characteristic for TA on the FTIR spectra of TA@IONP in Fig. 2b corroborates that TA adsorbed onto the IONP. The amount of TA required to coat the IONP that was estimated from surface area measurements depicted in Fig. S3 and Table S1,† as well as the TEM image in Fig. 2a, was lower than the amount of TA added to the dispersions containing the highest amount of IONP, *D*(10 : 0.5 : 1). It is possible that some of this excess TA also coated the TOCNF present in the hybrid foams.

**Fig. 3:**
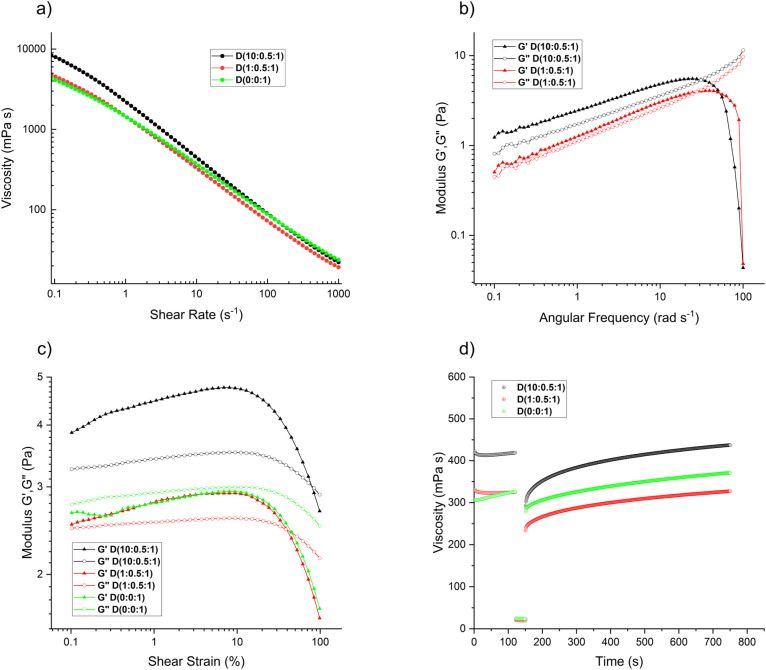
Rheological properties of *D*(10 : 0.5 : 1), *D*(1 : 0.5 : 1), and *D*(0 : 0 : 1) dispersions. (a) viscosity as a function of shear rate. (b) storage, *G*′, and loss, *G*′′ moduli *versus* angular frequency for dispersions obtained at 0.1% strain. (c) storage, *G*′, and loss, *G*′′ moduli *versus* shear strain for dispersions obtained at an angular frequency of 10 rad s^−1^. (d) 3ITT curves showing the time-dependent recovery of the viscosity at a shear rate of 10 s^−1^ after subjecting the dispersions to a constant rate of 1000 s^−1^ for 30 seconds (between 120 and 150 seconds).

Controlling the ice templating process is critical for producing foams with well-defined structures and mechanical integrity. The pH of the aqueous dispersions was adjusted to 7 to infer an electrostatic repulsion between the negatively charged TOCNF and TA@IONP and promote the formation of well-defined anisotropic structures after UIT/MFUIT. Foams fabricated from dispersions with pH values lower than 7 are less anisotropic because the agglomeration disrupts the ice growth and can cause the IONP to be trapped and thus less mobile when subjected to a magnetic field (Fig. S4†). The rheological properties at steady shear, oscillatory shear, and time-dependent viscosity recovery after strong shear (Fig. 3) have been conducted on dispersions that were vortexed vigorously for 5 minutes before measurements in order to mimic the conditions for UIT/MFUIT. Fig. 3a shows that TOCNF dominates the steady shear behavior of the dispersions except at very high amounts of IONP, *i.e. D*(10 : 0.5 : 1), where the viscosity at low shear rates increased. The frequency sweep measurements (Fig. 3b) show that the moduli remained below 10 Pa within the investigated frequency range. The addition of IONP resulted in a slight increase in the moduli, and the dispersion with the highest IONP content, *D*(10 : 0.5 : 1), displayed a weakly solid-like behavior at angular frequencies up to 31 rad s^−1^. The strain sweep measurement (Fig. 3c) corroborated that the dispersion with the highest amount of IONP, *D*(10 : 0.5 : 1), displayed the most solid-like behavior and that the TOCNF-only dispersion, *D*(0 : 0 : 1), showed a liquid-like behavior. The 3IIT measurements (Fig. 3d and Table S2†) show that the recovery of the TOCNF-only dispersion *D*(0 : 0 : 1) after subjecting the dispersion to high shear was fast compared to the IONP-containing dispersions. It is interesting to note that the high shear treatment inflicted a larger decrease of the initial viscosity for the *D*(1 : 0.5 : 1) dispersion compared to the *D*(0 : 0 : 1) dispersion, which suggests that a small amount of IONP promotes shear-induced structural disruption.

**Fig. 4:**
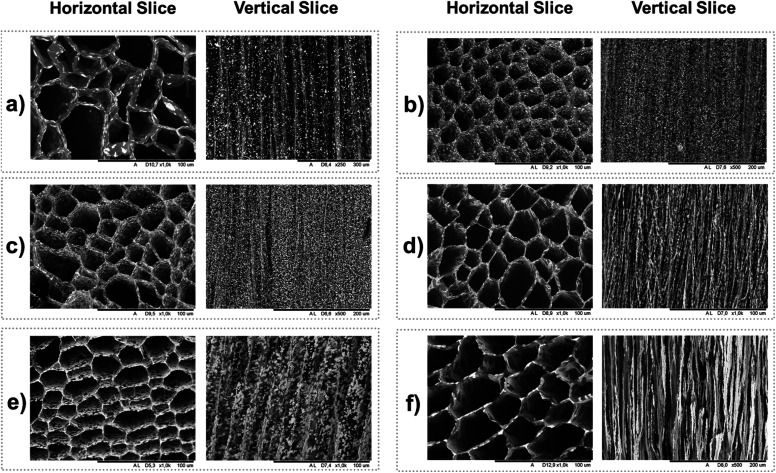
SEM images of horizontally and vertically sliced hybrid foams prepared by unidirectional ice-templating with (*F*_MFUIT_) and without (*F*_UIT_) application of a magnetic field. (a) *F*_UIT_(1 : 0 : 1), (b) *F*_UIT_(1 : 0.5 : 1) (c) *F*_UIT_(2.5 : 0.5 : 1), (d) *F*_MFUIT_(2.5 : 0.5 : 1), (e) *F*_UIT_(10 : 0.5 : 1) (f) *F*_MFUIT_(10 : 0.5 : 1). Scale bars are shown below each image.

The pore structures, anisotropy, the effect of the fabrication method, and the IONP distribution were studied using scanning electron microscopy. SEM images demonstrate that foams prepared by unidirectional ice-templating of dispersions with and without TA@IONP followed by freeze drying to remove the ice result in a columnar macropore structure (Fig. 4).^25^ Comparing the distribution of IONP (the bright spots) in the vertical slices of *F*_UIT_(1 : 0 : 1) (Fig. 4a) and *F*_UIT_(1 : 0.5 : 1) (Fig. 4b) clearly shows that coating the IONP with TA improved the distribution of IONP throughout the foam and reduced IONP aggregation. In addition, the SEM images with high magnification shown in Fig. S5† indicate that the presence of TA has contributed to a more uniform dispersion of IONP along the walls of foams fabricated using both UIT and MFUIT techniques. A comparison of Fig. 4c and d indicates that hybrid foams fabricated with magnetic field-enhanced unidirectional ice-templating (MFUIT) have a similar anisotropic columnar structure as the foams made without the use of a magnetic field (UIT). This suggests that the macroscopic anisotropic structure is primarily controlled by the growth of the ice crystals.^26^ The vertical slices (Fig. 4e) show that the IONP are randomly distributed and/or sometimes aggregated in the UIT foams whereas in the MFUIT foams (Fig. 4f) IONP form chains in the direction of the applied magnetic field and cover the walls. The effect of the magnetic field on the IONP structure was also investigated by TEM of IONP/TA/TOCNF dispersion drying on the TEM grid while subjected to a magnetic field (same as MFUIT), which also revealed chain formation (Fig. S6†). A comparison of Fig. 4d and f suggests that an increase in the amount of IONP resulted in the formation of some larger microclusters, but it should be noted that this effect had no discernible effect on the cellular structure of the foams.

**Fig. 5:**
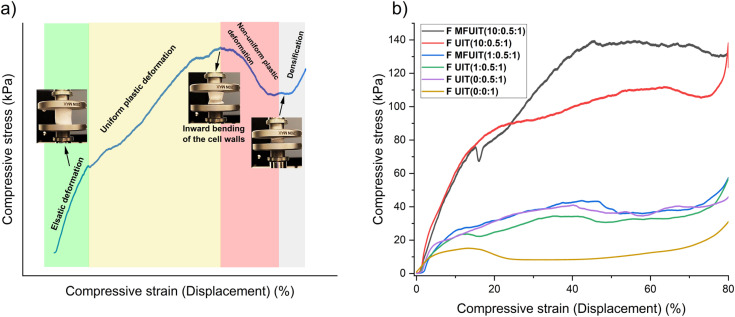
Compressive behavior of foams. (a) The elastic deformation region (green region), the uniform plastic deformation region (yellow region), the non-uniform plastic deformation region (red region) and foam densification region (grey region). (b) Stress–strain curve of TOCNF, TOCNF/TA foams, and foams with the highest (87 wt%) and the lowest (40 wt%) amount of IONP fabricated with UIT and MFUIT techniques.

**Fig. 6:**
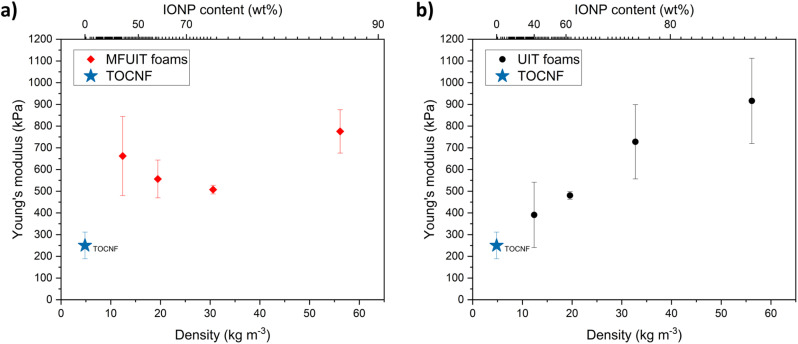
Compressive mechanical properties of hybrid IONP/TA/TOCNF foams. (a) Young's modulus of foams fabricated with MFUIT technique as a function of density and IONP content. (b) Young's modulus of foams fabricated with UIT technique as a function of density and IONP content. TOCNF foam has been shown with a star as a reference.

The compressive mechanical behavior of fabricated hybrid foams (Fig. 5a) can be divided into four regions: elastic, uniform plastic deformation, non-uniform plastic deformation, and foam densification. The images of the foams during increasing compression show that the cross-section decreased, which implies that foams behave as if they had been subjected to tensile rather than compressive stresses, as buckling is more common in compression. The behavior of *F*_MFUIT_(5 : 0.5 : 1) foam shown in Fig. 5a displayed an elastic deformation up to 10% strain and then deformed uniformly up to 44% strain, followed by a non-uniform deformation up to 53% strain at which the ultimate compressive strength is achieved. The compressive stress decreased as strain increased beyond 53% until the densification of the foam at 75% strain resulted in an increase in the compressive stress. The ultimate compressive strength and the strain at which the ultimate compressive strength was achieved were always higher for MFUIT compared to UIT foams (Table S3†). This could be an indication of structural reinforcement due to the even distribution of IONP onto TOCNF along the long axis and walls of the foams fabricated with the MFUIT technique. Surprisingly, the foams with the highest IONP content of 87 wt% (corresponding to *F*_UIT_(10 : 0.5 : 1) and *F*_MFUIT_(10 : 0.5 : 1)) were able to recover up to 14% of axial compression without permanent deformation. This shows that even a small relative amount of TOCNF is able to provide a flexible scaffold for the hybrid foams. The Young's modulus of the MFUIT (Fig. 6a) and UIT (Fig. 6b) hybrid foams ranged between 500 and 900 kPa. The UIT hybrid foams (Fig. 6b) displayed a linear increase in Young's modulus as a function of density, which suggests that the TA@IONP reinforced the foam walls. Surprisingly, the high Young's modulus for the MFUIT foam at a density of 12.4 mg cm^−3^ may indicate that the TA@IONP was able to form a percolated and stiff network at the TOCNF surface at these specific conditions. It should be noted that the amount of TOCNF is the same in all the foams and the density increase relate thus to an increasing amount of IONP in the hybrid foams. Furthermore, the fabrication technique had almost no effect on the density of the foams (Table S4†).

**Fig. 7:**
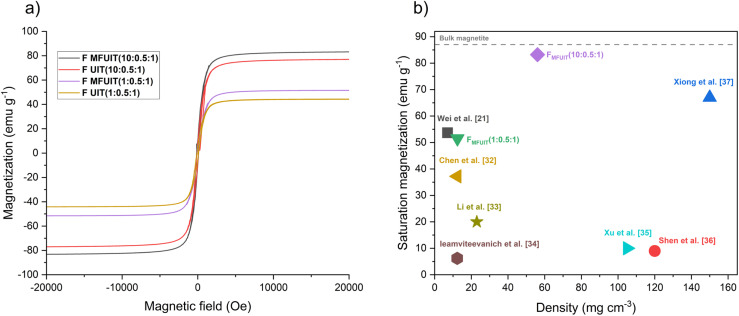
Magnetic properties of the hybrid foams. (a) Magnetic hysteresis loops of *F*_UIT_(1 : 0.5 : 1), *F*_MFUIT_(1 : 0.5 : 1), *F*_UIT_(10 : 0.5 : 1) and *F*_MFUIT_(10 : 0.5 : 1). (b) Comparison of saturation magnetization and density in hybrid foams with previously fabricated cellulose-IONP hybrid foams/aerogels with a density lower than 150 mg cm^−3^. The dashed line represents the saturation magnetization of bulk magnetite.

Fig. 7a shows that the foams display narrow magnetic hysteresis loops as well as low coercivity (*H*_c_) and low magnetic remanence (*M*_r_). The saturation magnetization (*M*_s_) normalized to the weight of the foams increased as the amount of IONP in the foams increased, which is consistent with previous studies.^27^ In comparison to the UIT foams, the MFUIT hybrid foams had higher *M*_s_ values and slightly higher *M*_r_ and *H*_c_ values (Table S5†). This can be explained by the fact that magnetic spins have a tendency to align in the direction of the applied magnetic field, which causes an induced magnetization.^28^ The *F*_MFUIT_(10 : 0.5 : 1) foam with an IONP content of 87 wt% displayed a saturation magnetization of 83.2 emu g^−1^, which is 95% of the value for bulk magnetite.^29,30^ It should be noted that it is necessary to add TA to fabricate stable hybrid foams, which unfortunately prohibited comparative magnetic measurements on foams that do not contain TA. However, previous works suggest that TA could result in a small decrease in *M*_s_ values due to the formation of a diamagnetic layer around the IONP.^31^ The saturation magnetization and density of the hybrid foams in this study were compared to previously fabricated cellulose-IONP hybrid foams/aerogels (Fig. 7b), indicating that low-density foams in this study have a *M*_s_ value comparable to previous works and that the intermediate-density (60 mg cm^−3^) hybrid foams with the highest IONP content (87 wt%) have a *M*_s_ value significantly higher than previously reported.^21,32–37^

## 4. Conclusions

Mechanically stable magnetic hybrid foams with an anisotropic structure have been successfully produced using unidirectional ice-templating and magnetic field-enhanced unidirectional ice-templating techniques. These foams have a density range of 12.4 to 56.1 mg cm^−3^ and an iron oxide nanoparticle (IONP) content ranging from 40 to 87 wt%, which is a significant increase in IONP content of lightweight magnetic hybrid foams when compared to previous studies. The foam fabricated with magnetic field-enhanced unidirectional ice-templating (MFUIT) with an IONP content of 87 wt% had a saturation magnetization of 83.2 emu g^−1^, which is equivalent to 95% of bulk magnetite. The electrostatic repulsion between the tannic acid (TA)-covered IONP (TA@IONP) and the TEMPO-oxidized cellulose nanofibril (TOCNF) promoted the formation of foams with a well-defined columnar foam structure and a more uniform distribution of IONP. Increasing the amount of TA@IONP in the foams resulted in an increase in Young's modulus, and enhancing the alignment of the TA@IONP by a magnetic field (MFUIT) also resulted in an increase in Young's modulus and the ultimate compressive strength. The preparation of hybrid anisotropic foams with a very high content of IONP could promote the development of novel materials for applications of magnetic foams.

## Author contributions

S. E. H., A. Y. and L. B. conceived the study. S. E. H. together with M. K., K. K. and A. Y. designed the experiments. S. E. H. prepared the materials and performed most of the characterization, and wrote the first draft of the manuscript. M. M. carried out the mechanical analysis. A. Å. carried out the AFM measurements and interpreted the data. S. E. H. and L. B. and M. H. S. iterated and improved the manuscript. All authors revised and commented on the manuscript.

## Conflicts of interest

There are no conflicts to declare.

## Supplementary Material

RA-013-D3RA01896B-s001
