# Supplementary material for: Highly magnetic hybrid foams based on aligned tannic acid-coated iron oxide nanoparticles and TEMPO-oxidized cellulose nanofibers
Source: RSC Adv. 2023 May 9;13(20):13919–27. doi: 10.1039/d3ra01896b (PMC10167729; doi:10.1039/d3ra01896b)
Supplement: RA-013-D3RA01896B-s001 [file RA-013-D3RA01896B-s001.pdf]

Electronic Supplementary Material (ESI) for RSC Advances.
This journal is © The Royal Society of Chemistry 2023

Highly magnetic hybrid foams based on aligned tannic acid-coated iron oxide nanoparticles and TEMPO-oxidized cellulose nanofibers

Seyed Ehsan Hadi[a,b], H. Aygül Yeprem[a,c], Agnes Åhl[a], Mohammad Morsali[a,b], Martin Kapuscinski[a], Konstantin Kriechbaum[a], Mika H. Sipponen[a,b], and Lennart Bergström[a]*.

[a]*Department of Materials and Environmental Chemistry, Stockholm University, Stockholm, 10691, Sweden.*
[b]*Wallenberg Wood Science Center, Department of Materials and Environmental Chemistry, Stockholm University, Stockholm, 10691, Sweden.*
[c]*Department of Metallurgical and Materials Engineering, Yıldız Technical University, Istanbul, 34220, Turkey.*
E-mail: lennart.bergstrom@mmk.su.se

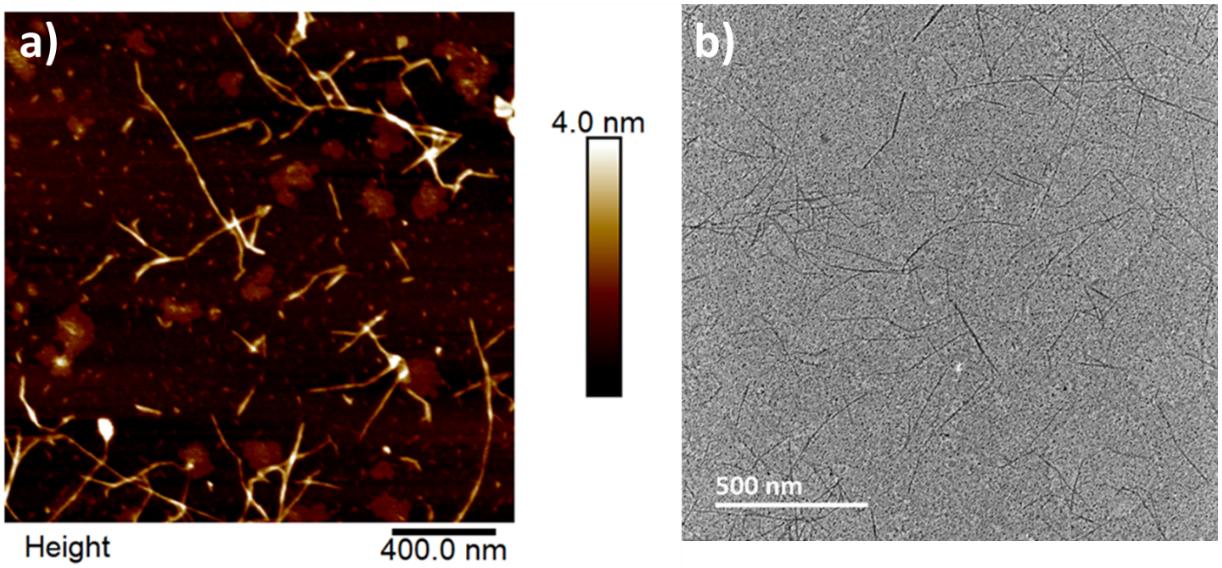

Fig. S1 Characterization of TOCNF. a) Peak-force tapping-mode AFM micrograph of TOCNF at the concentraion of 0.005 wt%; **b)** TEM image of TOCNF at the concentraion of 0.005 wt%.

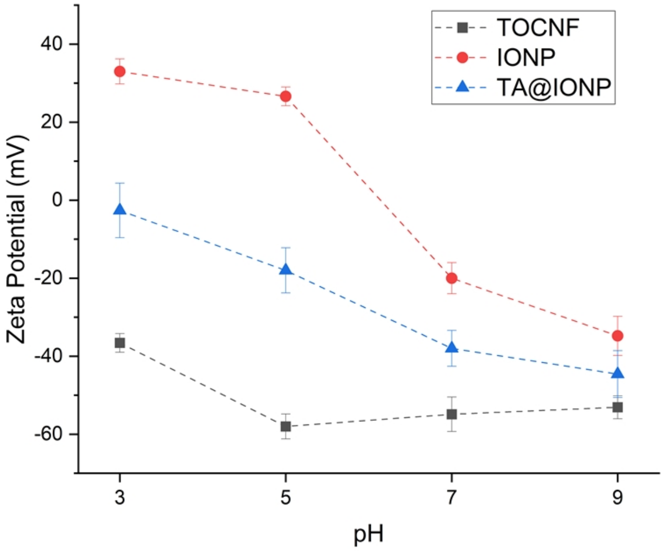

Fig. S2 Zeta potential values of TOCNF, IONP, and TA@ IONP at different pH levels.

Coating efficiency assessment of IONP with TA

N_2 adsorption/desorption isotherm of bare IONP were measured at a temperature of −196 °C (Fig. S3) and results were reported in Table S1. The BET surface area of bare IONP was analyzed to estimate the coating efficiency of TA. IONP theoretical surface area was calculated using assumptions based on their size (~190 nm), shape (spherical), and density (5100 mg cm^{-3}). The BET surface area of bare IONP (83.2 cm^2 mg^{-1}) was found to be higher than the theoretical surface area (65.8 cm^2 mg^{-1}), indicating that the IONP had some porosity that increased their surface area. The potential of the TA coating to not only fill the pores present on the IONP surface but also form a layer around them was considered. The coating efficiency of IONP with TA was also assessed using transmission electron microscopy (TEM), and the thickness of the TA layer was estimated to be about 5 nm (Fig. 2a).

The volume of TA (density, 2120 mg cm^{-3}) required to coat all the IONP with a 5 nm layer was calculated using the estimated value and the number of IONP in a given mass. The expected amount of TA required for coating 686.3 mg (mass used in BET analysis) of IONP was found to be 24.5 mg, and the result was adjusted by a factor of 83.2/65.8 to account for the higher BET surface area of IONP and the potential of TA to fill the pores. These calculations were applied to estimate the amount of TA needed to coat the IONP in foam samples with different concentrations of IONP (40 wt%, 165 mg and 87 wt%, 1650 mg of IONP). The amount of TA required, after adjustment, was found to be 7.47 mg and 74.74 mg for (1:0.5:1) foams and (10:0.5:1) foams, respectively. These values were lower than the amount of TA added to the dispersions (82.5 mg), indicating that there was excess TA in the system. It is possible that some of this excess TA also coated the cellulose nanofibrils (TOCNF) present in the hybrid foams.

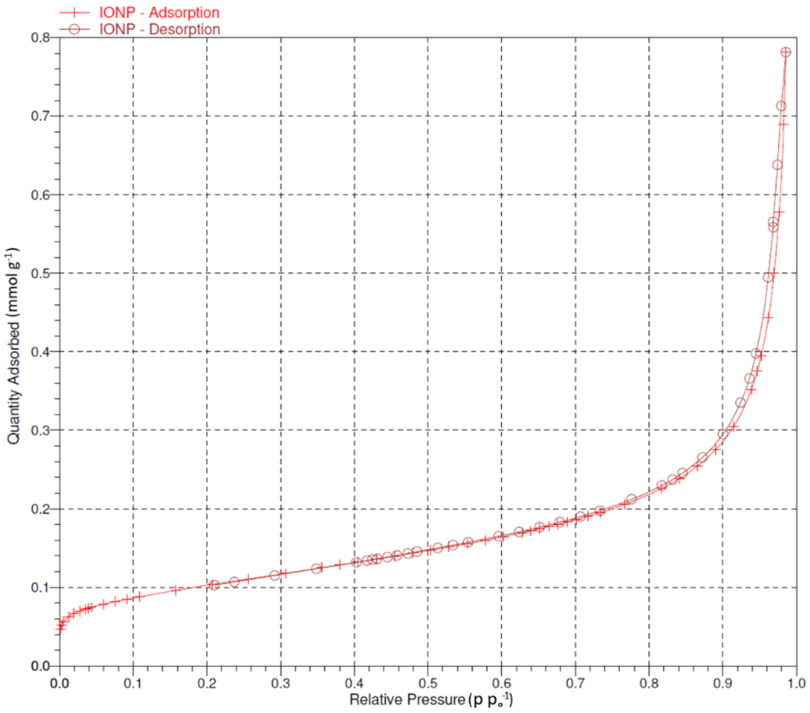

Fig. S3 N_2 adsorption/desorption isotherm plot of bare IONP.

Table S1 Results obtained from N_2 adsorption/desorption of bare IONP

BET Surface Area	8.326 m^2 g^{-1}
Single point adsorption total pore volume of pores less than 136.8327 nm diameter at p p_0^{-1} = 0.98	0.027 cm^3 g^{-1}
BJH Adsorption average pore diameter (4V/A)	14.38 nm
BJH Desorption average pore diameter (4V/A)	11.53 nm

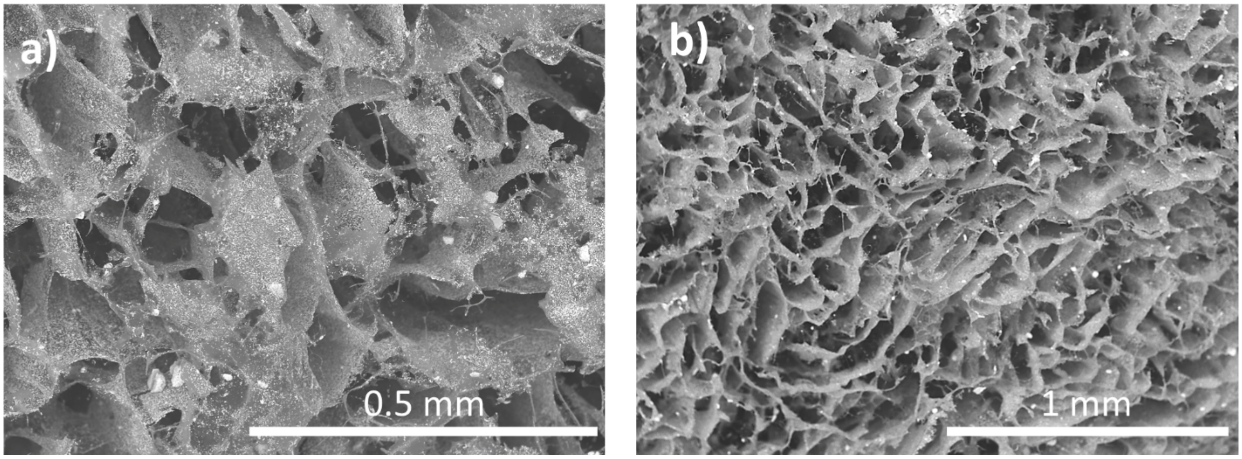

Fig. S4 SEM images of horizontally sliced F_{UIT}(2.5:0.5:1) foam processed with pH = 3 dispersion. Foams fabricated with highly viscous dispersions, such as those with pH = 3, are less anisotropic and have poor IONP distribution.

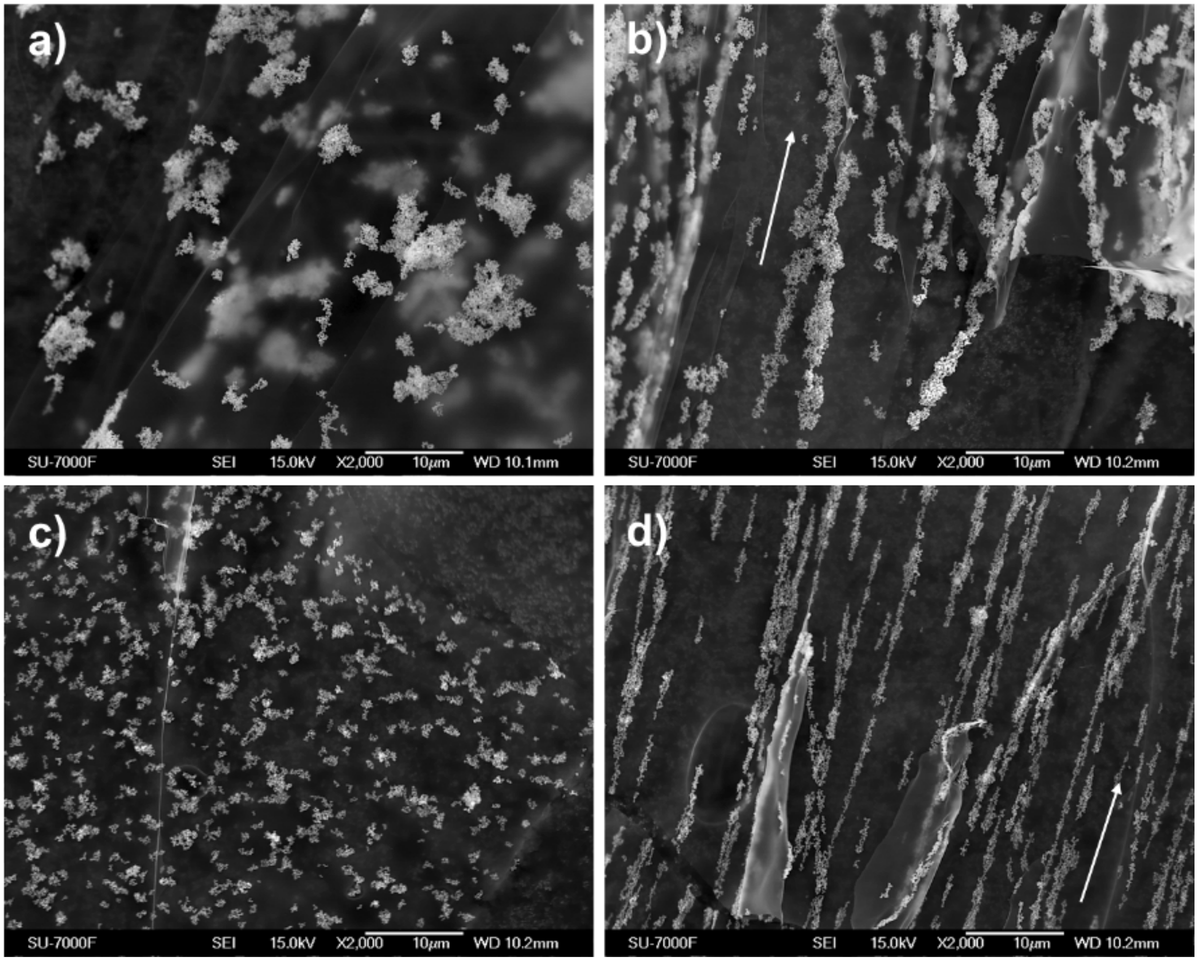

Fig. S5 High magnification SEM images of walls of hybrid foams; a) F_{UIT}(1:0:1) **b)** F_{MEUIT}(1:0:1) **c)** F_{UIT}(1:0.5:1) **d)** F_{MEUIT}(1:0.5:1). The arrows show the direction of the applied magnetic field.

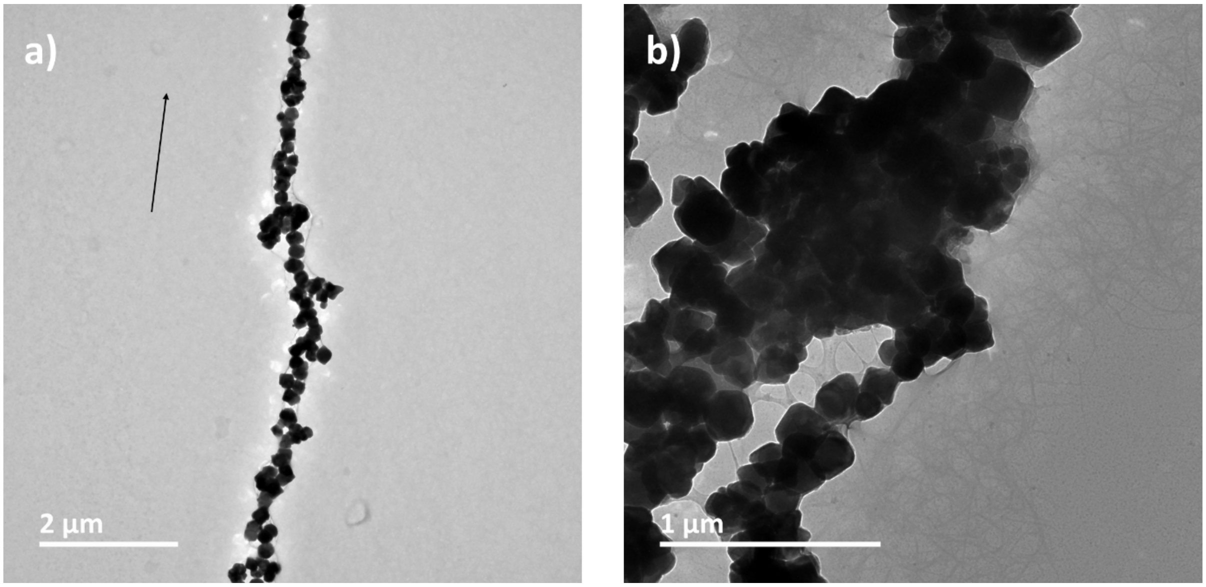

Fig. S6 TEM images of IONP/TA/TOCNF dispersion; a) subjected to a magnetic field (same as MFUIT) as it dries on a TEM gird, **b)** without magnetic field. The arrow shows the direction of the applied magnetic field.

Dispersion name	Viscosity recovery after 30 seconds (%)	Full viscosity recovery time (s)
D(10:0.5:1)	81.2	387
D(1:0.5:1)	79.8	553
D(0:0.5:1)	82.0	473
D(0:0:1)	92	153

Table S2 3ITT measurement results.

Table S3 Ultimate compressive strength of each foam and its strain at ultimate compressive strength.

Sample name	Ultimate compressive strength (kPa)	Strain at ultimate strength (%)
F_{UIT}(0:0:1)	15.2	13
F_{UIT}(0:1:1)	41.0	40
F_{UIT}(1:0:1)	18.3	24
F_{MEUIT}(1:0:1)	21.6	28
F_{UIT}(1:0.5:1	34.5	35
F_{MEUIT}(1:0.5:1)	43.6	42
F_{UIT}(2.5:0.5:1)	48.2	40
F_{MEUIT}(2.5:0.5:1)	57.3	61
F_{UIT}(5:0.5:1)	80.6	47
F_{MEUIT}(5:0.5:1)	89.5	53
F_{UIT}(10:0.5:1)	111.7	64
F_{MEUIT}(10:0.5:1)	137.2	70

Sample	IONP content (wt%)	Density (kg m^{-3})	Young's Modulus (kPa)	Specific Young's modulus (J g^{-1})	Toughness (kPa)	Specific Toughness (J g^{-1})
F_{UIT}(0:0:1)	0	4.82 ± 0.03	250.08 ± 61.23	51.94 ± 10.63	8.03 ± 0.72	1.66 ± 0.13
F_{UIT}(0:1:1)	0	7.39 ± 0.06	515.05 ± 188.54	69.83 ± 21.3	21.14 ± 1.53	2.86 ± 0.19
F_{UIT}(1:0:1)	50	9.54 ± 0.12	150.89 ± 31.74	15.84 ± 2.88	8.85 ± 0.6	0.93 ± 0.06
F_{MEUIT}(1:0:1)	50	9.56 ± 0.08	235.9 ±11.5	24.68 ± 1.15	8.41 ± 0.63	0.90 ± 0.06
F_{UIT}(1:0.5:1	40	12.38 ± 0.06	390.83 ± 150.56	31.60 ± 10.05	22.36 ± 4.1	1.81 ± 0.27
F_{MEUIT}(1:0.5:1)	40	12.4 ± 0.07	661.98 ± 182.12	53.44 ± 12.24	22.93 ± 0.62	1.85 ± 0.05
F_{UIT}(2.5:0.5:1)	62.5	19.57 ± 0.34	480.36 ± 17.61	24.56 ± 1.08	22.98 ± 5.21	1.17 ± 0.23
F_{MEUIT}(2.5:0.5:1)	62.5	19.48 ± 0.18	556.32 ± 87.05	28.58 ± 3.86	26.57 ± 4.65	1.36 ± 0.20
F_{UIT}(5:0.5:1)	77	32.74 ± 0.08	727.56 ± 171.05	22.23 ± 4.31	40.92 ± 3.81	1.25 ± 0.09
F_{MEUIT}(5:0.5:1)	77	30.59 ± 0.34	507.26 ± 20.03	16.59 ± 0.68	37.6 ± 3.9	1.23 ± 0.11
F_{UIT}(10:0.5:1)	87	56.17 ± 1.1	916.05 ± 196.05	16.36 ± 3.11	58.52 ± 2.73	1.04 ± 0.05
F_{MEUIT}(10:0.5:1)	87	56.15 ± 0.04	775.39 ± 99.99	13.81 ± 1.46	54.74 ± 17.5	0.97 ± 0.25

Table S4 Mechanical testing results.

Table S5 Magnetic hysteresis cycles measurement results.

Sample name	M_s (emu g^{-1})	H_c (Oe)	M_r (emu g^{-1})
F_{MEUIT}(10:0.5:1)	83.2	136	10.0
F_{UIT}(10:0.5:1)	77.0	91	8.0
F_{MEUIT}(1:0.5:1)	51.5	125	5.8
F_{UIT}(1:0.5:1)	44.3	104	5.2

Thermal stability of the foams

Fig. S7 illustrates the thermogravimetric analysis curve of foams. Thermal deterioration of CNFs, according to Zhang et al., can be classified into three zones.[1] The first zone is below 200 °C, which is mostly due to water loss from the sample and no significant loss from the bulk of the CNF. However, CNF in the second zone (200 °C to 380 °C) loses a substantial amount of weight followed by the third zone which the further thermal degradation such as graphitization may occur.[2] By calculating integral process decomposition temperature (IPDT) values using equation 1 to 3, the thermal stability of foams was established.[3,4]

$$IPDT(°C) = A^* K^* (T_f - T_i) + T_i \quad (1)$$

$$A^* = \frac{S_1 + S_2}{S_1 + S_2 + S_3} \quad (2)$$

$$K^* = \frac{S_1 + S_2}{S_1} \quad (3)$$

Table S6 Calculated IPDT values, moisture content, and the residual mass of each sample at 600 °C.

Sample Name	IPDT values (°C)	Moisture content (%)	Residual mass (%) at 600 °C
F_{UIT}(10:0.5:1)	8873	1.1	87.9
F_{UIT}(1:0.5:1)	1682	5	55.6
F_{UIT}(1:0:1)	1510	3.7	57.0
F_{UIT}(0:0.5:1)	713	8.5	30.2
F_{UIT}(0:0:1)	556	6.8	26.7

Comparing the IPDT values of foams (Table S6) reveals that the addition of TA enhanced the IPDT values, indicating that foams containing TA are more thermally stable than TOCNF-only foams. Furthermore, as expected, due to the intrinsic thermal stability of IONP, foams containing IONP have a much higher IPDT value as well as a significantly higher residual mass.

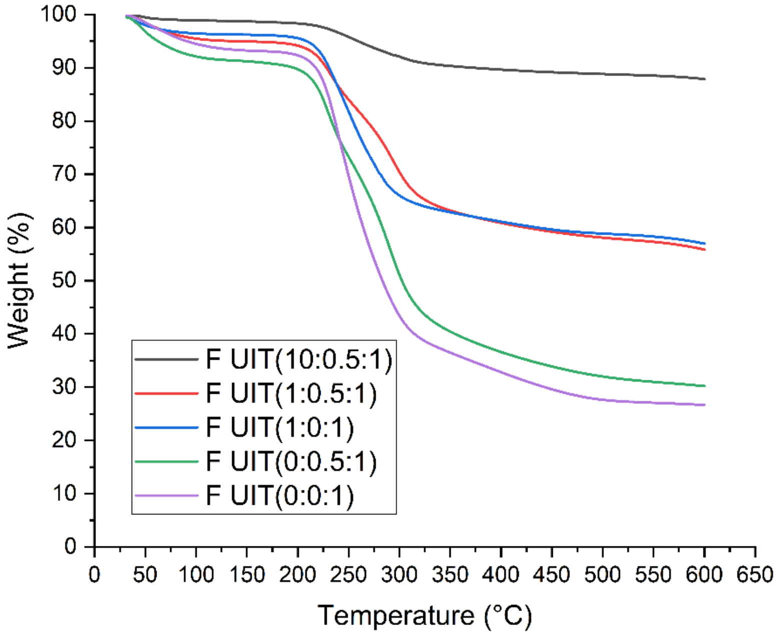

Fig. S7 Thermogravimetric analysis curve of hybrid foams.

References

1 N. Zhang, P. Tao, Y. Lu and S. Nie, *Cellulose*, 2019, **26**, 7823–7835.
2 F. Shafizadeh, *Journal of Analytical and Applied Pyrolysis*, 1982, **3**, 283–305.
3 M. Yadav, A. Sand and K. Behari, *International Journal of Biological Macromolecules*, 2012, **50**, 1306–1314.
4 M. Yadav, *Composites Communications*, 2018, **10**, 1–5.
